# Effect of Erythropoietin on Postresuscitation Renal Function in a Swine Model of Ventricular Fibrillation

**DOI:** 10.1155/2016/3567275

**Published:** 2016-10-25

**Authors:** Charalampos Pantazopoulos, Nicoletta Iacovidou, Evangelia Kouskouni, Paraskevi Pliatsika, Apostolos Papalois, Georgios Kaparos, Dimitrios Barouxis, Panagiotis Vasileiou, Pavlos Lelovas, Olympia Kotsilianou, Ioannis Pantazopoulos, Georgios Gkiokas, Clara Garosa, Gavino Faa, Theodoros Xanthos

**Affiliations:** ^1^Department of Anaesthetics, Liver Transplant Unit, King's College Hospital, London, UK; ^2^Neonatology Department, Aretaieio Hospital, Medical School, National and Kapodistrian University of Athens, Athens, Greece; ^3^Department of Biopathology, Aretaieio Hospital, Medical School, National and Kapodistrian University of Athens, Athens, Greece; ^4^MSc “Cardiopulmonary Resuscitation”, Medical School, National and Kapodistrian University of Athens, Athens, Greece; ^5^ELPEN Research-Experimental Centre, Athens, Greece; ^6^2nd Department of Surgery, Medical School, National and Kapodistrian University of Athens, Athens, Greece; ^7^Division of Pathology, Department of Surgical Sciences, University of Cagliari Medical School, Cagliari, Italy; ^8^School of Medicine, European University Cyprus, Nicosia, Cyprus

## Abstract

*Purpose*. To investigate the effect of EPO administration on postresuscitation renal function.* Methods*. Twenty-four female Landrace/Large-White piglets aged 10–15 weeks with average weight of 19 ± 2 kg were randomly assigned to 2 different groups of 12 subjects each. After the end of an 8-minute ventricular fibrillation, the control group (Group C) received saline as placebo, whereas the EPO group (Group E) received EPO 5000 U/kg. The animals were resuscitated according to the 2010 European Resuscitation Council Guidelines for Resuscitation.* Results*. Five animals (41.67%) from Group C and 11 animals (91.67%) from Group E achieved ROSC (*p* = 0.027). Eight animals (66.67%, 5 surviving and 3 nonsurviving) from Group C suffered severe kidney damage or AKI compared to animals from Group E, in which none of the swine had evidence of severe kidney damage or AKI (*p* = 0.001). There was a statistically significant difference in all tested biochemical markers between the two groups, as well as a positive correlation of creatinine with NGAL, L-FABP, and IL-18 (summed mean values' *p* = 0.049, 0.01, and 0.004, resp.).* Conclusions*. Administration of EPO protected swine from postresuscitation acute kidney injury.

## 1. Introduction

High-quality cardiopulmonary resuscitation (CPR) optimizes forward blood flow after cardiac arrest. However, the compression-related cardiac output is 15–30% of its prearrest values. As the kidney is very susceptible to hemodynamic perturbations, the intra-arrest and postcardiac ischemia and hypoxemia are main factors for acute kidney injury (AKI) [1].

Acute kidney injury is the sudden and sustained reduction in renal function, which causes a constant accumulation of nitrogenous and nonnitrogenous products and toxins, fluid disorders of rapid onset, and electrolyte and acid-base imbalance. During the last decade, various novel biochemical markers for the early detection of AKI have been identified. These are kidney-produced proteins, such as the neutrophil gelatinase-associated lipocalin (NGAL) and cytokine interleukin-18 (IL-18), as well as low molecular weight substances, such as the Liver-Type Fatty-Acid Binding Protein (L-FABP) [2–4].

Erythropoietin (EPO) is an endogenous glycoprotein and member of the family of type I cytokines. Although exogenous EPO is commonly used in daily clinical practice to treat anemia in patients with chronic kidney failure [5], research has shown that EPO can reduce apoptosis and inflammation, while it promotes angiogenesis, antioxidant activity, and blood oxygen carrying capacity, thus exerting cardioprotective and neuroprotective effects [6, 7]. Although EPO receptors have been identified in various tissues [8], there are no data regarding their protective effects on renal function after cardiac arrest. The aim of the present study was to investigate the effect of EPO administration on renal function in an established model of cardiac arrest and resuscitation.

## 2. Methods

### 2.1. Study Design

The objective of this observational animal study was to investigate the effect of EPO administration on renal function in an established model of cardiac arrest and resuscitation. The study was conducted according to the Utstein style guidelines for uniform reporting of laboratory CPR research. The experimental protocol was approved by the Directorate of Veterinary Services of Prefecture of Athens, Attica, Greece, according to Greek legislation regarding ethical and experimental procedures (protocol number 23/10-01-2012).

### 2.2. Animal Subjects and Handling

The study used 24 female Landrace/Large-White piglets aged 10–15 weeks with average weight of 18–20 kg, all supplied from the same breeder (Validakis, Athens, Greece). The animals were fasted overnight but had free access to water.

### 2.3. Study Protocol

The protocol has been described in detail elsewhere [9]. Briefly, initial sedation was achieved by intramuscular injection of ketamine hydrochloride (10 mg/kg), midazolam (0.5 mg/kg), and atropine (0.05 mg/kg). The animals were transferred to the operating table and anesthesia was induced with an IV bolus dose of propofol (2 mg/kg) via the marginal auricular vein. The pigs were then intubated with a 4.0–5.0 mm cuffed endotracheal tube which was secured on the lower jaw, and successful intubation was ascertained with auscultation of both lungs while ventilating with a self-inflating bag. The animals were then immobilized in the supine position on the operating table.

The animals were mechanically ventilated with a volume-controlled ventilator with a tidal volume of 15 mL/kg and fraction of inspired oxygen (FiO2) of 0.21. The respiratory frequency was adjusted to maintain an arterial partial pressure of carbon dioxide between 35 and 40 mmHg. A bolus dose of cisatracurium (0.15 mg/kg) was administered to ascertain synchrony with the ventilator. Continuous infusion of propofol 150 *μ*g/kg/min or more, if needed, was used to maintain adequate anesthetic depth and fentanyl 4 *μ*g/kg to ensure satisfactory analgesia. Cardiac rhythm and heart rate were monitored by electrocardiography (ECG), using leads I, II, III, aVR, aVL, and aVF. Pulse oximetry was monitored continuously.

Right carotid artery and right internal jugular vein were surgically prepared and catheterized under aseptic conditions. Aortic pressures were measured using a fluid-filled catheter (model 6523, USCI CR, Bart, Papapostolou, Athens, Greece) advanced via the right carotid artery into the thoracic aorta. Mean arterial pressure (MAoP) was determined by electronic integration of the aortic blood pressure waveform. A catheter was inserted into the right atrium via the right jugular vein for continuous measurement of right atrial pressures. Coronary perfusion pressure (CPP) was electronically calculated as the difference between minimal aortic diastolic pressure (DAoP) and the simultaneously measured right atrial diastolic pressure. The second internal jugular vein was also surgically prepared, and a 5 F flow-directed pacing catheter (Pacel, 100 cm; St. Jude Medical, Ladakis, Athens, Greece) was advanced into the apex of the right ventricle. All catheters were calibrated before use, and their correct position was verified by the presence of the typical pressure waveform.

### 2.4. Experimental Protocol

After surgery, the animals were allowed a 30 min stabilization period before baseline data were collected. Before the experimental procedure, the piglets were randomly assigned to 2 different groups of 12 subjects each, according to the agents used, by means of a sealed envelope.

Ventricular fibrillation was induced with a 9 V ordinary cadmium battery via a pacing wire forwarded into the right ventricle through the exposed right jugular vein, as previously described [10], and was confirmed by ECG and a sudden drop in MAoP. Mechanical ventilation and administration of anesthetics were discontinued simultaneously with the onset of VF, and the animals were left untreated for 8 min.

After the end of the 8th minute, the control group (Group C) received saline as placebo (10-mL dilution, bolus), whereas the EPO group (Group E) received EPO 5000 U/kg. All drugs were injected via the marginal auricular vein, followed by a 10 mL saline flush to assist faster circulation of medications. The researchers were blinded to EPO or saline, respectively, until the experiment was completed and all hemodynamic and survival data were collected.

The animals were resuscitated according to the 2010 European Resuscitation Council Guidelines for Resuscitation immediately after EPO or saline administration [11]. Mechanical ventilation was resumed with 100% oxygen, and automatic continuous precordial compressions were initiated. LUCAS CPR device (LUCAS, Jolife, Lund, Sweden) provided high-quality chest compressions at a rate of 100/min and a depth of 5 cm, while the quality of the given CPR (rate and depth of chest compressions) was assessed before and during each experiment to ensure uniformity. After 2 min of CPR, defibrillation was attempted with a 4 J/kg monophasic shock (Primedic Defi-B Defibrillator; Metrax GmbH, Rottweil, Germany); CPR was resumed for another 2 min after each defibrillation attempt. A bolus dose of adrenaline (0.02 mg/kg) was administered after the third shock, while further bolus doses of adrenaline (0.02 mg/kg) were administered every fourth minute during CPR.

Successful resuscitation was defined as return of spontaneous circulation (ROSC) with a MAoP of at least 60 mmHg for a minimum of 5 minutes. After ROSC, the animals were monitored closely and mechanically ventilated for 6 h under general anesthesia at the prearrest settings, while blood samples were collected at 2, 4, and 6 hours after ROSC for quantification of creatinine (creatinine, Abbott Diagnostics, Architect Analyzer, Germany) and NGAL (Pig NGAL 044 Elisa Kit, Bioporto Diagnostics, Denmark), as well as urine L-FABP (Human L-FABP HK404 Elisa Kit, cross-reactivity with swine, Hycult Biotech, The Netherlands) and IL-18 (Pig IL-18 Platinum Elisa Kit, eBioscience, Austria). No other interventions (drugs, cardioversion, or defibrillation attempts) were made after ROSC. Subsequently, anesthesia was discontinued, all catheters were removed as previously described, and manual ventilation was initiated [12]. Atropine 0.2 mg/kg followed by neostigmine 0.05 mg/kg was administered when spontaneous swallowing reflex was detected, whereas extubation was performed after adequate inspiration depth was confirmed. Each animal was then transferred to the animal house for observation for 48 hours, while blood and urine samples were collected at 24 and 48 hours after ROSC. The surviving animals were humanely euthanized by an intravenous overdose of pentobarbital and underwent necropsy.

Thoracic and abdominal organs were examined for gross evidence of traumatic injuries or other pathologies. A kidney sample, including the ureter, was collected in the hilar region along the lower kidney diameter in both kidneys, in all animals. Tissue samples were formalin-fixed, routinely processed, and paraffin-embedded. Five micron-thick sections were stained with H&E and PAS method for evidencing the brush border of the proximal tubules [13]. The histological analysis of each kidney included the capsule, the subcapsular zone, proximal and distal tubules, the afferent and efferent arteries, the vessels at the corticomedullary limit, the Henle loops, the collecting tubules, and the renal papillae.

### 2.5. Data Analysis

Statistical analysis of the data was performed using Statistical Package for the Social Sciences version 15.0 (SPSS Inc., Chicago, IL, USA) and Stata statistical software package version 9.2 (StataCorp LP, College Station, TX, USA). Fisher's exact test was used to investigate associations between group and ROSC percentages. Due to small number of subjects, the nonparametric Wilcoxon-Mann–Whitney test for independent samples was utilized for comparisons of quantitative measurements between the two groups at each distinct time-point, either during CPR or after ROSC. Spearman's rho nonparametric coefficient of correlation was utilized for investigating direct correlations between quantitative measurements. We further utilized clustered regression analysis for longitudinal data to examine overall effect of parameters on repeated measurements. A cut-off point of *p* value <0.05 was used to mark statistical significance; however all *p* values are reported.

Required sample size, with power of at least 80% at *a* = 5% level of statistical significance, was computed at 12 subjects per group (C, E) in order to demonstrate a clinically important difference between controls, which were expected to suffer severe kidney damage or AKI at a percentage of at least 70%, and EPO group, which were expected to suffer severe kidney damage or AKI at a percentage of 10% or lower.

## 3. Results

A significant difference was observed in ROSC between the 2 groups, as 5 animals (41.67%) from Group C and 11 animals (91.67%) from Group E achieved ROSC (*p* = 0.027). Time to ROSC was 5.60 ± 0.894 min in Group C and 2.90 ± 1.375 in Group E (*p* = 0.005). The mean number of shocks during CPR was 2.80 ± 0.447 in Group C and 1.45 ± 0.687 in Group B (*p* = 0.005), while no shocks were delivered after ROSC. Total dose of epinephrine administered during CPR in Group C and Group E was 0.80 ± 0.447 and 0.09 ± 0.301 mg (*p* = 0.006), respectively.

Although no statistically significant differences were observed in baseline and 8-minute untreated VF hemodynamic parameters between the 2 groups, significant hemodynamic differences were observed between groups after the onset of CPR (Table 1). All animals that were successfully resuscitated were monitored for 6 hours, during which significant differences in hemodynamic parameters were observed (Table 2).

**Figure 1:**
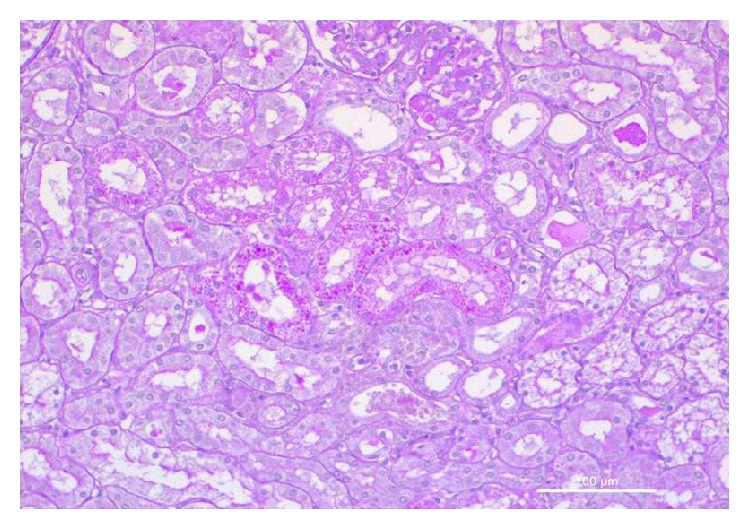
Acute kidney injury: in the center, tubular cells undergoing apoptosis show multiple PAS-positive globules (Group C).

**Figure 2 fig2:**
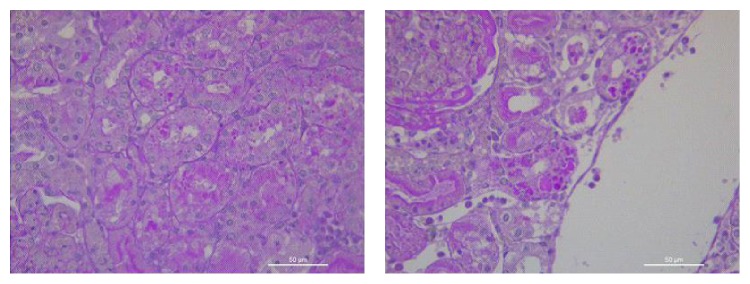
Acute kidney injury: apoptosis of tubular cells is better evidence at higher power (Group C).

**Figure 3:**
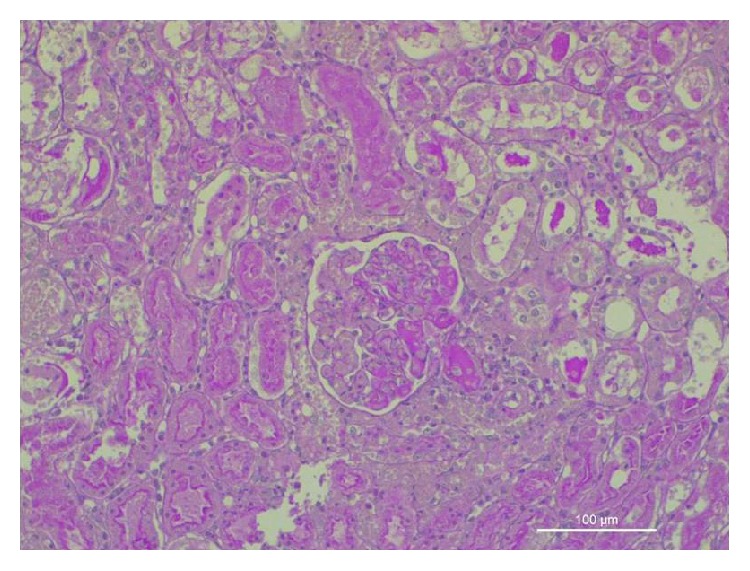
Acute kidney injury: segmental glomerular necrosis associated with thrombosis of the afferent artery (Group C).

At histology, multiple morphological changes were detected both in the control animals (Group C) and in animals treated with EPO (Group E). At low power, renal pathological changes appeared often subtle and complex. The most relevant pathological changes were detected in renal tubules, the most severe lesions being detected in proximal tubules. The spectrum of cell injury ranged from loss of cell adhesion, characterized at histology by a simple enlargement of the intercellular spaces, to severe coagulative necrosis involving large segments of affected nephrons. In less affected kidneys, pathological tubular changes were segmental, being difficult to detect at low power. Moreover, pathological changes in tubular epithelial cells might be seen as a continuum, ranging from subtle focal changes of the brush border to complete destruction of the cell structure. Many morphological changes were detected in animals of both groups, including loss of the brush border of proximal tubular cells, detachment of adjacent tubular cells, dilatation of the tubular lumen, vacuolization of the cytoplasm and of tubular cells, dedifferentiation of the proximal and distal tubular epithelium, necrosis of individual tubular cells, and tubular cell apoptosis. Decapitation, that is, the loss of the brush border in proximal tubular cells, was the most frequent pathological lesion detected in the kidney of both groups. Only two lesions were significantly more severe in the kidney of control group animals, as compared to the EPO-treated group animals: apoptosis of tubular epithelial cells, often appearing as small apoptotic globules that were highlighted by PAS staining method (Figures 1 and 2); complete loss of the brush border in a large number of proximal tubular epithelial cells. Glomerular changes, mainly represented by features of focal segmental glomerular sclerosis, suggestive of the presence of podocyte pathology (Figure 3) were exclusively observed in Group C animals.

According to the tubular changes detected, we categorized AKI into two types, mild and severe. Mild AKI was diagnosed when the loss of the brush border was present alone, or in association with vacuolization. Severe AKI was diagnosed in all cases in which tubular cell death was observed, sometimes in association with tubular dilatation and/or cellular casts. Significant differences regarding kidney injury were noticed histologically between Group C and Group E subjects, regarding both all participating subjects and only ROSC-gaining subjects (Table 3, Figures 1 [Fig fig2]–3). Eight animals (66.67%, 5 surviving and 3 nonsurviving) from Group C suffered severe kidney damage or AKI compared to animals from Group E, in which none of the swine had evidence of severe kidney damage or AKI (*p* = 0.001) (Table 3). In our study, biomarkers at baseline did not differ significantly between the 2 groups. There was a statistically significant difference in all tested biochemical markers between the two groups (Table 4), as well as a positive correlation of creatinine with NGAL, L-FABP, and IL-18 (summed mean values *p* = 0.049, 0.01, and 0.004, resp.) (Table 5). Although we found a correlation between the hemodynamic parameters and the biochemical markers (Table 6), this was not as strong as the correlation between biochemical markers and histologically confirmed renal injury when examined concurrently with hemodynamic parameters (Table 7), based on statistical significance.

## 4. Discussion

While research on postcardiac arrest syndrome focuses on myocardial dysfunction and brain injury, several studies indicate that AKI is common in cardiac arrest survivors, with rates of postcardiac arrest AKI ranging from 40 to 80% [14–19]. These differences may be due to using different definitions of AKI [20], as well as the effect of preexisting pathology or complications, such as the development of sepsis and nephrotoxic agents [18]. In addition, postresuscitation AKI occurs in different causes of cardiac arrest, although asphyxia has more severe kidney injury and gets worse prognosis [21].

The pleiotropic actions of EPO are indicated by the identification of its receptor in nonhaematopoietic cells and tissues including neurons, astrocytes, microglia, and endothelial cells, as well as cells of myocardium and kidney, while the putative mechanisms involved in EPO-induced cardioprotection are related to its antiapoptotic, anti-inflammatory, and angiogenic effects [22, 23]. The most important finding in our study was the protective effect of EPO on postresuscitation renal function. Although creatinine, NGAL, L-FABP, and IL-18 increased during the postresuscitation period in both groups, they were significantly greater in Group C, while none of the animals of Group E suffered histologically confirmed AKI. Also, in our study, EPO administration improved hemodynamics both during CPR and after ROSC, which may be partly responsible for the good renal function in Group E. Our research group has shown that EPO administration as a single bolus dose of 5000 U/kg immediately before the initiation of resuscitative efforts enhances both DAoP and CPP during CPR, thus limiting resuscitative period and improving ROSC [24]. Similarly, Grmec et al. reported that EPO administered intravenously within the first 2 minutes of CPR after out-of-hospital cardiac arrest facilitates ROSC, intensive care unit admission, 24-hour survival, and hospital survival [25]. Early ROSC decreases the severity of postcardiac arrest myocardial stunning and promotes postresuscitation hemodynamic stability, which may improve renal perfusion and function [26].

In addition, EPO administration may increase systemic vasoconstriction and possess direct vasoconstrictive effects in isolated renal resistance vessels which may also preserve adequate renal perfusion after ROSC [27]. On the other hand, treatment with EPO promptly downregulates circulating levels of renin and aldosterone [28, 29]. Although the EPO-induced inhibition of the renin-angiotensin-aldosterone system may decrease glomerular filtration rate, it decreases renal afferent arteriolar constriction and improves renal microcirculation, thus increasing oxygen tension in the renal environment [27, 29]. In addition, EPO optimizes oxygen delivery to tissues by modulating regional blood flow; the protective effects are mediated by binding of EPO to a heteromeric receptor complex consisting of two *β*-common receptors and two EPO receptors [30].

Although AKI is characterized by death of the tubular epithelium and activation and expansion of the tubulointerstitium with inflammatory cells, while extensive apoptosis and necrosis may exist, there is clear evidence that administration of EPO at or near the time of injury significantly improves recovery acutely via inhibition of apoptosis, stimulation of antioxidant and angiogenic action, and suppression of proinflammatory cytokine mediators [31–34]. Our histological data strongly support this hypothesis. In this study, apoptosis of tubular cells was much more frequent and diffuse in control animals, as compared to kidneys of EPO-treated animals. Moreover, EPO treatment appeared to partially protect tubular cells from the loose of their brush border, confirming the hypothesis of a protective activity of EPO on tubular cell function and integrity. A new interesting finding emerging from our study is the presence of glomerular changes in the kidney of control animals and their absence in the kidney of EPO-treated animals. This finding first confirms that every animal model of acute kidney injury differs in several significant ways from other experimental models of acute tubular necrosis and from AKI in man [35, 36]. Moreover, the finding of features of focal segmental glomerular sclerosis (Figure 3) suggests a previously unreported glomerular involvement in our model of AKI and suggests the hypothesis regarding a possible protective role for EPO in the protection of podocyte changes. Administration of EPO in both in vitro and in vivo models of ischemic AKI significantly expedited renal structural and functional recovery [37]. The aforementioned results have been confirmed in porcine models; EPO administration during renal ischemia and reperfusion provided significant protection against functional impairment [38]. Considering that the region immediately surrounding damage is typically relatively deficient in endogenous EPO, administration of exogenous EPO can provide increased tissue protection. However, effective use of EPO as therapy for tissue injury requires higher doses than for haematopoiesis [30]. Interestingly, murine studies have shown that administration of EPO (5,000 U/kg) at the time of ischemia-reperfusion injury significantly decreases tubular cell apoptosis, particularly in the region of the hypoxia-sensitive proximal straight tubule, while enhancing tubular regeneration and decreased cast formation [39]. Collectively, the aforementioned data indicate that administration of EPO 5000 U/kg appears to be superior compared to lower doses following renal I/R [40]. It has to be mentioned that, in contrast to myocardial cells, the renal epithelial cells are considered stable and have the ability to enter the cell cycle for regeneration and repair if stimulated [41]. Nevertheless, the EPO-mediated improvement in hemodynamics may compensate the lack of regeneration of myocardial cells, thus decreasing postcardiac arrest myocardial dysfunction [31].

Serum creatinine concentration does not change until around 50% of kidney function is lost, which increases the risk for missing a therapeutic opportunity and eventually mortality [42]. Diagnosis of AKI after cardiac arrest may be delayed due to fluid resuscitation which often results in a positive fluid balance, potentially diluting the serum creatinine concentration. Pickering et al. reported that creatinine concentration decreased during six hours of fluid infusion at 1 litre per hour in simulated patients, irrespective of fluid type or extent of change in GFR, which delayed diagnosis of AKI by 2 to 9 hours [43]. In our study, abnormal creatinine levels were noticed after 24 hours. As unchanged plasma creatinine in the first 24 hours after cardiac arrest may signify renal injury, the conclusions of Pickering et al., who reported that creatinine sampling should be delayed at least one hour following a large fluid bolus to avoid dilution [43], may not apply within the first 24 hours after ROSC.

Of note, in our study there was positive correlation of creatinine with NGAL, L-FABP, and IL-18, all of which increased at 2 hours after ROSC. An increase in NGAL within a few hours of cardiac arrest has been suggested to indicate AKI, even in creatinine-negative patients [17, 31]. Considering that NGAL increases early after ROSC, its high levels may be strongly predictive of both AKI and survival-to-hospital discharge [44]. In cardiac surgical patients, however, only L-FABP has been reported as a useful biomarker for early detection of AKI. This is due to the pattern of increase of NGAL and L-FABP after cardiac surgery which results from differences in the mechanism of urinary secretion. L-FABP is rapidly upregulated and secreted from damaged proximal tubular cells after AKI, while NGAL is filtered by glomeruli and reabsorbed by proximal tubules, taking a relatively longer period to increase compared with the L-FABP level [45].

Moreover, NGAL may increase its usefulness in the diagnosis of postresuscitation AKI when combined with IL-18 and L-FABP and if a curve of plasma values rather than a single plasma measurement is determined [46]. Interleukin-18 is produced by macrophages and other cell types present in the kidney and contributes to the renal damage observed during ischemia-reperfusion injury [47]. Although IL-18 mediates ischemic acute tubular necrosis, its levels in urine concentration have only moderate diagnostic value for the early detection of AKI [48]. In our study, however, IL-18 increased within 2 hours after ROSC and was associated with postresuscitation AKI, a finding which strengthens current data regarding its diagnostic role. In a similar way, L-FABP increased within 2 hours after ROSC, a finding which confirms the results of other authors [49, 50]. Urinary L-FABP has a large dynamic range and could monitor the different levels of postresuscitation AKI [51]. Also, as renal tubule epithelial cells are rich in mitochondria, they are quite vulnerable under hypoxic conditions. During cardiac arrest, hypoxia injures the outer medullary region and decreases peritubular capillary blood flow, which is further aggravated by the reperfusion-induced oxidative stress after ROSC [52]. Considering that urinary L-FABP is stable in urine, our results indicate that it may be a marker of decreased postresuscitation peritubular capillary blood flow, reflecting the postcardiac arrest hypoxic condition and AKI [53].

Considering that the time window between renal insult and development of AKI in postcardiac arrest patients with myocardial dysfunction and/or severe hemodynamic instability can be varied in different patients and AKI often is diagnosed too late, NGAL, L-FABP, and IL-18 seem promising biomarkers for early detection of AKI. Of note, the correlation between biochemical markers and histologically confirmed renal injury was stronger than the correlation between the hemodynamic parameters and the biochemical markers. However, before full adoption in clinical practice can be accomplished, adequately powered clinical trials are strongly warranted.

*Limitations*. Our study has several limitations. The relatively small sample size is one limitation of our study. However, the statistically significant differences were detected between the groups. In addition, cardiac arrest was induced by ventricular fibrillation in our study; therefore, our results may not apply in nonshockable rhythms. Moreover, the use of anesthesia is possible to yield independent myocardial or brain protective effects which may have influenced our results. Our study was performed on healthy animals, which does not resemble to the possible scenario in humans. Also, we were unable to measure end-tidal carbon dioxide during the experiment. Finally, we used only one dose of EPO; thus we are unable to comment whether different dosage may have exerted different or no effect.

## 5. Conclusions

In our study, administration of EPO protected swine from postresuscitation AKI. Taking into account its pleiotropic effects, use of EPO during the periarrest period merits further research.

## Supplementary Material

In our study, we found no significant differences in baseline hemodynamics between the two groups.

## Figures and Tables

**Table 1:** Hemodynamic variables presented as mean (±SD) values, between the two groups during cardiopulmonary resuscitation.

Variable	CPR 2′ min			CPR 4′ min			CPR 6′ min			CPR 8′ min		
	Group C	Group E	*p* value	Group C	Group E	*p* value	Group C	Group E	*p* value	Group C	Group E	*p* value
	*N* = 12	*N* = 12		*N* = 12	*N* = 5		*N* = 11	*N* = 2		*N* = 2	*N* = 0	
SAoP (mmHg)	58.3 (±7.98)	73.6 (±5.26)	<0.001	59.4 (±9.31)	69.0 (±4.84)	0.045	60.3 (±11.50)	66.5 (±9.19)	0.373	50.5 (±6.36)	NA	NA
DAoP (mmHg)	27.8 (±9.65)	46.2 (±13.53)	0.010	31.5 (±8.52)	40.8 (±9.09)	0.020	32.5 (±7.64)	40.5 (±17.67)	0.381	26.0 (±2.82)	NA	NA
MAoP (mmHg)	37.9 (±8.73)	55.3 (±10.60)	0.001	40.7 (±8.49)	50.0 (±7.51)	0.023	41.8 (±8.31)	49.5 (±14.84)	0.322	34.0 (±4.24)	NA	NA
CPP (mmHg)	18.1 (±10.34)	36.3 (±14.29)	0.011	20.5 (±9.09)	30.8 (±11.30)	0.081	20.9 (±9.09)	26.0 (±15.55)	0.487	13.5 (±3.53)	NA	NA

CPR = cardiopulmonary resuscitation, SAoP = systolic arterial pressure, DAoP = diastolic arterial pressure, MAoP = mean arterial pressure, CPP = coronary perfusion pressure, and NA = nonapplicable.

**Table 2:** Hemodynamic variables presented as mean (±SD) values, between the two groups after return of spontaneous circulation.

Variable	ROSC 1 min			ROSC 2 h			ROSC 4 h			ROSC 6 h		
	Group C	Group E	*p* value	Group C	Group E	*p* value	Group C	Group E	*p* value	Group C	Group E	*p* value
	*N* = 5	*N* = 11		*N* = 5	*N* = 11		*N* = 5	*N* = 11		*N* = 5	*N* = 11	
SAoP (mmHg)	112.6 (±20.65)	98.7 (±6.84)	0.192	109.8 (±9.73)	111.7 (±9.07)	0.570	102.0 (±12.74)	103.0 (±6.57)	0.733	100.4 (±15.10)	108.4 (±3.55)	0.569
DAoP (mmHg)	63.4 (±3.04)	64.5 (±6.84)	0.609	63.6 (±3.97)	73.8 (±4.19)	0.004	70.6 (±4.27)	76.5 (±3.98)	0.026	71.0 (±5.43)	79.0 (±3.91)	0.015
MAoP (mmHg)	79.8 (±8.81)	76.0 (±6.76)	0.459	79.0 (±5.78)	86.3 (±5.80)	0.047	81.0 (±6.78)	85.4 (±4.43)	0.256	80.8 (±8.49)	88.8 (±3.34)	0.030
CPP (mmHg)	56.2 (±5.58)	56.5 (±7.63)	0.776	56.2 (±6.22)	66.5 (±4.01)	0.005	63.0 (±5.33)	69.0 (±3.72)	0.031	63.6 (±5.77)	72.3 (±3.82)	0.017
HR (bpm)	185.6 (±32.99)	150.6 (±14.66)	0.047	177.0 (±33.29)	150.3 (±9.87)	0.061	161.4 (±12.66)	133.5 (±15.05)	0.003	157.8 (±7.39)	128.0 (±9.15)	0.002

ROSC = return of spontaneous circulation, SAoP = systolic arterial pressure, DAoP = diastolic arterial pressure, MAoP = mean arterial pressure, CPP = coronary perfusion pressure, HR = heart rate.

**Table 3:** Number and frequency (%) of levels of biochemically and histologically confirmed renal injury among subjects and comparisons between control versus EPO and ROSC versus non-ROSC subjects.

Parameter	Total	Group C	Group E	*p value*
ROSC	16 (66.67)	5 (41.67)	11 (91.67)	***0.027***

Renal injury (ROSC subjects)				
None	3 (18.75)^*∗*^	0 (0.00)^*∗∗*^	3 (27.27)	***<0.001***
Mild	3 (18.75)	0 (0.00)	3 (27.27)	
Moderate	5 (31.25)	0 (0.00)	5 (45.45)	
Severe	0 (0.00)	0 (0.00)	0 (0.00)	
AKI	5 (31.25)	5 (100.00)	0 (0.00)	

AKI (ROSC subjects)	5 (31.25)	5 (100.00)^*∗∗*^	0 (0.00)	***<0.001***

Severe injury or AKI (ROSC subjects)	5 (31.25)	5 (100.00)	0 (0.00)	***<0.001***

ROSC = return of spontaneous circulation; AKI = acute kidney injury.

^*∗*^0.01 < *p* value ≤ 0.05; ^*∗∗*^ *p* value ≤ 0.001, for differences in AKI frequencies of ROSC versus non-ROSC subjects (within either total of subjects or same treatment group, accordingly); differences marked with asterisks next to “none”; kidney damage category frequencies correspond to overall frequency differences for all kidney damage categories (i.e., not only specific differences for “none”).

**Table 4:** Biochemical measurements and comparisons for ROSC subjects.

Parameter	All subjects	Group C	Group E	*p value* ^*∗*^
	(*N* = 16)	(*N* = 5)	(*N* = 11)	
	Mean (SD)	Mean (SD)	Mean (SD)	
Creatinine (mg/dL)				
Baseline	0.58 (0.088)	0.58 (0.109)	0.59 (0.083)	*0.761*
2 hours	0.70 (0.081)	0.76 (0.054)	0.67 (0.078)	***0.048***
4 hours	0.78 (0.065)	0.84 (0.054)	0.75 (0.052)	***0.018***
6 hours	0.86 (0.051)	0.86 (0.054)	0.85 (0.052)	*0.843*
24 hours	1.93 (0.614)	2.76 (0.054)	1.51 (0.087)	***0.002***
48 hours	3.44 (1.053)	5.27 (0.152)	2.89 (0.166)	***0.010***
Mean^*∗∗*^	1.43 (0.372)	1.77 (0.462)	1.28 (0.196)	***0.014***

NGAL (ng/mL)				
Baseline	133.29 (15.893)	129.01 (16.686)	135.25 (15.941)	*0.394*
2 hours	596.89 (104.449)	743.47 (24.163)	530.26 (22.464)	***0.002***
4 hours	524.25 (92.649)	653.77 (15.744)	465.38 (24.026)	***0.002***
6 hours	439.83 (130.717)	624.03 (8.372)	356.10 (30.400)	***0.002***
24 hours	373.33 (160.136)	591.57 (6.239)	264.22 (13.568)	***0.002***
48 hours	294.61 (160.889)	576.43 (12.360)	210.06 (7.952)	***0.011***
Mean^*∗∗*^	457.53 (132.065)	643.39 (11.222)	373.06 (31.474)	***<0.001***

L-FABP (ng/mL)				
Baseline	1.40 (0.188)	1.45 (0.222)	1.38 (0.177)	*0.954*
2 hours	1.91 (0.158)	2.11 (0.008)	1.82 (0.101)	***0.002***
4 hours	2.52 (0.256)	2.88 (0.033)	2.35 (0.034)	***0.002***
6 hours	2.73 (0.277)	3.10 (0.003)	2.56 (0.113)	***0.002***
24 hours	2.41 (0.229)	2.72 (0.064)	2.26 (0.029)	***0.002***
48 hours	2.22 (0.182)	2.54 (0.035)	2.12 (0.018)	***0.011***
Mean^*∗∗*^	2.37 (0.223)	2.68 (0.022)	2.22 (0.047)	***<0.001***

IL-18 (ng/mL)				
Baseline	0.09 (0.006)	0.09 (0.006)	0.09 (0.006)	*0.908*
2 hours	0.10 (0.005)	0.10 (0.002)	0.09 (0.002)	***0.001***
4 hours	0.11 (0.003)	0.11 (0.002)	0.10 (0.001)	***0.002***
6 hours	0.12 (0.014)	0.14 (0.002)	0.11 (0.002)	***0.002***
24 hours	0.16 (0.039)	0.21 (0.003)	0.13 (0.002)	***0.002***
48 hours	0.12 (0.018)	0.15 (0.002)	0.11 (0.002)	***0.010***
Mean^*∗∗*^	0.12 (0.016)	0.14 (0.003)	0.11 (0.002)	***<0.001***

ROSC = return of spontaneous circulation.

^*∗*^Comparisons at distinct time-point between groups utilize Wilcoxon-Mann–Whitney nonparametric test for independent samples; comparisons regarding overall (mean) measurements utilize clustered regression models for longitudinal data, including all measurements after baseline (2–48 hours).

^*∗∗*^Mean measurements refer to all time-points summed (2–48 hours), apart from baseline values.

**Table 5:** Correlations between creatinine and other biochemical markers for ROSC subjects (*N* = 16).

Parameter	Spearman's rho for correlation with creatinine	*p* value
NGAL		
Baseline	0.906	**<0.001**
2 hours	0.874	**<0.001**
4 hours	0.844	**<0.001**
6 hours	0.506	**0.045**
24 hours	0.979	**<0.001**
48 hours	0.972	**<0.001**
Mean^*∗*^	0.498	**0.049**

L-FABP		
Baseline	0.793	**<0.001**
2 hours	0.789	**<0.001**
4 hours	0.717	**0.002**
6 hours	0.478	0.061
24 hours	0.966	**<0.001**
48 hours	0.973	**<0.001**
Mean^*∗*^	0.623	**0.010**

IL-18		
Baseline	0.868	**<0.001**
2 hours	0.750	**0.001**
4 hours	0.744	**0.001**
6 hours	0.509	**0.044**
24 hours	0.964	**<0.001**
48 hours	0.905	**<0.001**
Mean^*∗*^	0.773	**0.004**

^*∗*^Mean measurements refer to all time-points summed (2–48 hours), apart from baseline values.

**Table 6:** Biochemical markers and effect of hemodynamic measurements (per unit increase) for ROSC subjects (*N* = 16).

Parameter^*∗*^	Effect of hemodynamic measurement on biochemical marker^*∗∗*^	*p value *
	*b*-coefficient (95% C.I.)	
Creatinine (mg/dL)		
CPR: SAoP (per mmHg)	−0.004 (−0.030 to 0.023)	*0.780 *
CPR: DAoP (per mmHg)	−0.016 (−0.032 to 0.001)	*0.070*
CPR: MAoP (per mmHg)	−0.015 (−0.035 to 0.004)	*0.106 *
CPR: CPP (per mmHg)	−0.014 (−0.029 to 0.002)	*0.082*
ROSC: SAoP (per mmHg)	0.029 (0.015 to 0.044)	***0.001***
ROSC: DAoP (per mmHg)	−0.018 (−0.060 to 0.023)	*0.360*
ROSC: MAoP (per mmHg)	0.023 (−0.023 to 0.069)	*0.306 *
ROSC: CPP (per mmHg)	−0.011 (−0.048 to 0.026)	*0.541*

NGAL (ng/mL)		
CPR: SAoP (per mmHg)	−12.730 (−19.455 to −6.006)	***0.001***
CPR: DAoP (per mmHg)	−8.317 (−14.341 to −2.293)	***0.010***
CPR: MAoP (per mmHg)	−10.293 (−17.009 to −3.577)	***0.005***
CPR: CPP (per mmHg)	−7.808 (−13.295 to −2.322)	***0.008***
ROSC: SAoP (per mmHg)	6.789 (−1.845 to 15.424)	*0.114 *
ROSC: DAoP (per mmHg)	−20.694 (−30.126 to −11.263)	***<0.001***
ROSC: MAoP (per mmHg)	−3.240 (−23.362 to 16.883)	*0.736 *
ROSC: CPP (per mmHg)	−13.899 (−22.804 to −4.994)	***0.005***

L-FABP (ng/mL)		
CPR: SAoP (per mmHg)	−0.021 (−0.033 to −0.009)	***0.002***
CPR: DAoP (per mmHg)	−0.014 (−0.024 to −0.004)	***0.011***
CPR: MAoP (per mmHg)	−0.017 (−0.029 to −0.006)	***0.006***
CPR: CPP (per mmHg)	−0.013 (−0.022 to −0.004)	***0.010***
ROSC: SAoP (per mmHg)	0.011 (−0.004 to 0.026)	*0.139 *
ROSC: DAoP (per mmHg)	−0.036 (−0.051 to −0.021)	***<0.001***
ROSC: MAoP (per mmHg)	−0.006 (−0.041 to 0.028)	*0.693 *
ROSC: CPP (per mmHg)	−0.025 (−0.039 to −0.010)	***0.002***

IL-18 (ng/mL)		
CPR: SAoP (per 10 mmHg)^†^	−0.013 (−0.021 to −0.005)	***0.004***
CPR: DAoP (per 10 mmHg)^†^	−0.010 (−0.017 to −0.003)	***0.010***
CPR: MAoP (per 10 mmHg)^†^	−0.012 (−0.020 to −0.004)	***0.006***
CPR: CPP (per 10 mmHg)^†^	−0.009 (−0.016 to −0.003)	***0.010***
ROSC: SAoP (per 10 mmHg)^†^	0.010 (−0.001 to 0.020)	*0.059*
ROSC: DAoP (per 10 mmHg)^†^	−0.023 (−0.034 to −0.012)	***0.001***
ROSC: MAoP (per 10 mmHg)^†^	−0.001 (−0.026 to 0.023)	*0.910*
ROSC: CPP (per 10 mmHg)^†^	−0.015 (−0.026 to −0.005)	***0.008***

^*∗*^Comparisons regarding overall measurements utilize clustered regression models for longitudinal data, with summed hemodynamic measurements being the dependent variables and (repeatedly measured) biochemical markers being the independent variable (i.e., effect of mean hemodynamic measurements on biochemical markers); each row corresponds to separate model regarding the effect of specific hemodynamic measurement on specific biochemical marker.

^*∗∗*^Hemodynamic measurements refer to all time-points summed as mean, according to protocol period (2–8 minutes for CPR, 0–120 minutes for ROSC), apart from baseline.

^†^Effects refer to per 10 mmHg change in mean hemodynamic measurements, due to very small *b*-coefficients.

**Table 7:** Biochemical measurements and concurrent effect of hemodynamic measurements (per unit increase) and histopathological group (presence of AKI) for ROSC subjects (*N* = 16).

Parameter^*∗*^	Effect of hemodynamic measurement^*∗∗*^	*p value *	Effect of AKI presence	*p value *
	*b*-coefficient (95% C.I.)		*b*-coefficient (95% C.I.)	
Creatinine (mg/dL)				
CPR: SAoP (per mmHg)	0.025 (−0.001 to 0.051)	***0.012***	0.675 (0.377 to 0.974)	***<0.001***
CPR: DAoP (per mmHg)	−0.001 (−0.006 to 0.005)	*0.860 *	0.513 (0.112 to 0.913)	***0.016***
CPR: MAP (per mmHg)	0.005 (−0.006 to 0.015)	*0.369 *	0.560 (0.177 to 0.944)	***0.007***
CPR: CPP (per mmHg)	0.001 (−0.005 to 0.007)	*0.799*	0.526 (0.119 to 0.933)	***0.015***
ROSC: SAoP (per mmHg)	0.020 (0.005 to 0.034)	***0.011***	0.381 (0.037 to 0.725)	***0.032***
ROSC: DAoP (per mmHg)	0.027 (−0.019 to 0.072)	*0.228*	0.641 (0.293 to 0.989)	***0.001***
ROSC: MAP (per mmHg)	0.027 (−0.003 to 0.058)	*0.077*	0.537 (0.234 to 0.840)	***0.002***
ROSC: CPP (per mmHg)	0.018 (−0.015 to 0.050)	*0.270 *	0.599 (0.260 to 0.939)	***0.002***

NGAL (ng/mL)				
CPR: SAoP (per mmHg)	−1.461 (−3.430 to 0.507)	*0.134*	264.050 (243.929 to 284.171)	***<0.001***
CPR: DAoP (per mmHg)	−0.461 (−1.568 to 0.647)	*0.389 *	268.321 (249.770 to 286.873)	***<0.001***
CPR: MAP (per mmHg)	−0.739 (−2.115 to 0.637)	*0.270 *	266.447 (246.608 to 286.285)	***<0.001***
CPR: CPP (per mmHg)	−0.538 (−1.612 to 0.536)	*0.303 *	267.324 (247.535 to 287.113)	***<0.001***
ROSC: SAoP (per mmHg)	−0.231 (−1.077 to 0.616)	*0.570 *	274.768 (253.272 to 296.265)	***<0.001***
ROSC: DAoP (per mmHg)	−2.155 (−4.935 to 0.625)	*0.119 *	263.286 (240.501 to 286.071)	***<0.001***
ROSC: MAP (per mmHg)	−0.997 (−2.778 to 0.785)	*0.252 *	272.461 (252.644 to 292.278)	***<0.001***
ROSC: CPP (per mmHg)	−1.180 (−3.288 to 0.929)	*0.252 *	267.717 (248.147 to 287.287)	***<0.001***

L-FABP (ng/mL)				
CPR: SAoP (per mmHg)	−0.001 (−0.005 to 0.002)	*0.396 *	0.449 (0.416 to 0.481)	***<0.001***
CPR: DAoP (per mmHg)	−0.001 (−0.003 to 0.002)	*0.573 *	0.450 (0.417 to 0.483)	***<0.001***
CPR: MAP (per mmHg)	−0.001 (−0.004 to 0.002)	*0.501 *	0.449 (0.417 to 0.480)	***<0.001***
CPR: CPP (per mmHg)	−0.001 (−0.003 to 0.002)	*0.676 *	0.452 (0.420 to 0.484)	***<0.001***
ROSC: SAoP (per mmHg)	−0.001 (−0.003 to 0.001)	*0.380 *	0.464 (0.418 to 0.511)	***<0.001***
ROSC: DAoP (per mmHg)	−0.005 (−0.012 to 0.002)	*0.141 *	0.434 (0.383 to 0.485)	***<0.001***
ROSC: MAP (per mmHg)	−0.003 (−0.007 to 0.002)	*0.239*	0.456 (0.414 to 0.497)	***<0.001***
ROSC: CPP (per mmHg)	−0.004 (−0.009 to 0.002)	*0.191*	0.441 (0.397 to 0.485)	***<0.001***

IL-18 (ng/mL)				
CPR: SAoP (per 10 mmHg)^†^	0.001 (−0.001 to 0.004)	*0.295*	0.034 (0.031 to 0.038)	***<0.001***
CPR: DAoP (per 10 mmHg)^†^	−0.001 (−0.002 to 0.001)	*0.407*	0.033 (0.030 to 0.037)	***<0.001***
CPR: MAP (per 10 mmHg)^†^	−0.001 (−0.002 to 0.001)	*0.848*	0.034 (0.030 to 0.037)	***<0.001***
CPR: CPP (per 10 mmHg)^†^	−0.001 (−0.002 to 0.001)	*0.805*	0.034 (0.030 to 0.037)	***<0.001***
ROSC: SAoP (per 10 mmHg)^†^	0.001 (−0.001 to 0.003)	*0.160*	0.033 (0.030 to 0.036)	***<0.001***
ROSC: DAoP (per 10 mmHg)^†^	0.001 (−0.004 to 0.005)	*0.725*	0.034 (0.030 to 0.038)	***<0.001***
ROSC: MAP (per 10 mmHg)^†^	0.001 (−0.002 to 0.005)	*0.399*	0.034 (0.031 to 0.037)	***<0.001***
ROSC: CPP (per 10 mmHg)^†^	0.001 (−0.003 to 0.004)	*0.755*	0.034 (0.031 to 0.037)	***<0.001***

AKI presence = Group C.

^*∗*^Comparisons regarding overall measurements utilize clustered regression models for longitudinal data, with summed hemodynamic measurements and AKI presence being the dependent variables and (repeatedly measured) biochemical markers being the independent variable (i.e., effect of mean hemodynamic measurements and AKI presence on biochemical markers); each row corresponds to model defined by separate hemodynamic measurement specified, along with presence of AKI (the two being covariates), on specific biochemical marker mentioned.

^*∗∗*^Hemodynamic measurements refer to all time-points summed as mean, according to protocol period (2–8 minutes for CPR, 0–120 minutes for ROSC), apart from baseline.

^†^Effects refer to per 10 mmHg change in mean hemodynamic measurements, due to very small *b*-coefficients.
