# Supplementary material for: Effect of Erythropoietin on Postresuscitation Renal Function in a Swine Model of Ventricular Fibrillation
Source: Biomed Res Int. 2016 Oct 25;2016:3567275. doi: 10.1155/2016/3567275 (PMC5099488; doi:10.1155/2016/3567275)
Supplement: Supplementary file 1 — In our study, we found no significant differences in baseline hemodynamics between the two groups. [file 3567275.f1.pdf]

Supplemental Table. Baseline data

	Group C	Group E	p value
SAoP (mmHg)	112.34 ± 25.60	101.34 ± 15.64	0.176
DAoP (mmHg)	83.42 ± 19.39	81.45 ± 12.00	0.392
MAoP (mmHg)	93.06 ± 21.46	88.08±14.35	0.290
CPP (mmHg)	75.23±9.86	73.54±8.77	0.362
HR	122.2 ± 27.46	131.55 ± 46.5	0.630

SAoP = systolic arterial pressure, DAoP = diastolic arterial pressure, MAoP = mean arterial pressure, CPP = coronary perfusion pressure, HR= Heart rate
